# Antennas as Precise Sensors for GNSS Reference Stations and High-Performance PNT Applications on Earth and in Space

**DOI:** 10.3390/s21124192

**Published:** 2021-06-18

**Authors:** Stefano Caizzone, Miriam Schönfeldt, Wahid Elmarissi, Mihaela-Simona Circiu

**Affiliations:** Institute of Communications and Navigation, German Aerospace Center (DLR), 82234 Wessling, Germany; miriam.schoenfeldt@dlr.de (M.S.); wahid.elmarissi@dlr.de (W.E.); mihaela-simona.circiu@dlr.de (M.-S.C.)

**Keywords:** GNSS, antenna, multipath, PNT, reference station, satellite, precise positioning

## Abstract

Satellite navigation is more and more important in a plethora of very different application fields, ranging from bank transactions to shipping, from autonomous driving to aerial applications, such as commercial avionics as well as unmanned aerial vehicles (UAVs). In very precise positioning, navigation, and timing (PNT) applications, such as in reference stations and precise timing stations, it is important to characterize all errors present in the system in order to account possibly for them or calibrate them out. Antennas play an important role in this respect: they are indeed the “sensor” that capture the signal in space from global navigation satellite systems (GNSS) and thereby strongly contribute to the overall achievable performance. This paper reviews the currently available antenna technologies, targeting specifically reference stations as well as precise GNSS antennas for space applications, and, after introducing performance indicators, summarizes the currently achievable performance. Finally, open research issues are identified, and possible approaches to solve them are discussed.

## 1. Introduction

The use of satellite navigation is nowadays very widespread and embraces almost all fields of modern life [1,2,3,4]. The availability of multiple constellations and new signals in the last decades enables a strong improvement in the achievable accuracy in positioning and timing. Progress in the receiver technology has also made it possible to achieve extraordinary results at lower prices, making it accessible to a wider range of users [5].

While mass-market applications have been the main driver in the technology development, GNSS still strongly rely and depend on ground reference stations for monitoring the signals sent by the satellites, as well as identifying anomalies and characterization of imperfections that can later be communicated to (professional) users, for instance, by means of augmentation systems [1].

Technological advances for reference stations were apparently stronger in the 1980–1990s when the number of reference stations was growing, and therefore, the business side of it was more appealing. In the latest years, however, a revived interest in improving the technology of reference stations can be recorded, most probably due to the addition of new frequencies and new constellations in the GNSS world, with the corresponding need for updating the stations.

Furthermore, at the present stage, new scenarios are being developed, such as reference stations not placed on Earth but, for instance, orbiting Earth in low orbit (LEO) in order to achieve even better monitoring without the limitations of atmosphere and ground multipath [6].

The scope of this paper is to analyze state-of-the-art technologies in terms of antennas for GNSS in very high-performance applications, taking reference stations, both on the ground and in space, as exemplary applications. In particular, we investigate how far commercially available antennas are from what an “ideal antenna” should be and evaluate the imperfections and their impact on the overall system performance.

Starting from big and canonically used antennas for ground reference stations, it is also investigated in what respect performance degrades when using more compact (while still high-end) antennas that could, for instance, be used as “mobile reference stations,” both on Earth and in space.

In order to evaluate and compare the performance of the different state-of-the-art antennas, performance indicators are defined in Section 2, considering the different domains in which an antenna influences the GNSS measurement: gain roll-off, multipath suppression capability, group delay, and phase center variations are investigated.

Section 3 shows state-of-the-art performance for ground reference station antennas, while Section 4 covers high-end antennas for space applications. Section 5 then exemplarily shows the GNSS measured performance of some of the antennas. Conclusions are drawn in Section 6.

## 2. Performance Parameters and the “Ideal Antenna”

Antennas for reference stations have to act as sensors to monitor the signals emitted by the satellites. Therefore, ideally, antennas should be totally transparent, i.e., capable of properly collecting the signal, without adding any distortion (in terms of amplitude, delay, etc.) to the signal [7].

Such an ideal antenna would have an omnidirectional pattern in the upper hemisphere, and it would then very sharply decrease below the horizon to not receive any signals from the lower hemisphere (e.g., from multipath). Moreover, it would be able to collect signals at all bands of interest, and its transfer function would be very constant over frequency and angle.

As a matter of fact, real antennas do not manage to achieve this ideal behavior. Due to various design techniques, approximations thereof can, however, be achieved.

In order to identify the critical parameters of an antenna for GNSS, its operation, together with the receiver, is analyzed. The calculation of position, velocity, and time (PVT) in a GNSS receiver starts from two kinds of measurement: the pseudorange measurement and carrier phase measurement [1]. The former calculates the delay in time between the signal emission at the satellite side and the signal collection at the receiver, while the second calculates the distance (in an ambiguous way) by measuring the phase at which the signal is received.

A good signal-to-noise ratio is needed in order to be able to perform the measurements in an accurate way. Moreover, it shall also be avoided that non-line-of-sight (NLOS) propagation disturbs the calculation of the distance between satellite and receiver, i.e., multipath shall be minimized [8].

In view of the above-mentioned functional needs, the relevant parameters of a receiving antenna can be summarized as follows:

1. Gain (roll-off, uniformity), affecting the amount of received power and the signal to noise level;
2. Group delay variation (GDV), affecting the pseudorange measurement;
3. Phase center variation (PCV), affecting the carrier phase measurement;
4. Multipath suppression capability, estimated through multipath suppression indicators (MPSIs), related to the amount of crosspolar radiation.

### 2.1. Gain Roll-Off and Uniformity

The gain of the antenna affects the amplitude of the signal available at the receiver in multiple ways. On the one hand, it is important to ensure that the signals are available at the receiver with enough power to be clearly distinguishable from noise after correlation [2]. This can be obtained by using active antennas, i.e., antennas with LNAs integrated into the antenna itself, with the purpose of strongly reducing the impact of noise of the further components (splitters, cables, etc.) between antenna and receiver, in case the two are connected through long cables or splitters. High-performing antennas should, moreover, have a uniform gain over both angle and frequency, such that the antenna itself does not introduce variations in the received signal. This uniformity is usually obtained by antenna designs using multiple feeds and hence capable of exciting very pure and uniform gain patterns [9].

In order to be able to receive signals from satellites over a wide range of elevations, high-performing antennas shall also have a gain pattern with a very broad beamwidth (defined as the angular region where the gain level is not lower than a given threshold, usually 3 or 10 dB, with respect to the maximum gain). This requirement contradicts, however, the need for the pattern to drop sharply below the horizon (to reduce the influence of reflectors). The fulfillment of both requirements, at the same time, is hardly achievable in real life, unless very big structures are used, such as the ones adopted in ground-based augmentation system (GBAS) ground reference antennas [10]. Small antennas usually have a broader beamwidth with a scarce suppression of the pattern below the horizon, while more sophisticated antennas manage to strongly suppress back lobes at expenses of beamwidth, i.e., gain at low elevations.

The amount of gain drop from the zenith to the horizon is usually termed as “gain roll-off” [7]. As later discussed in this paper, gain roll-off is usually stronger (about 10–20 dB) for antennas with multipath-limiting structures (i.e., for which the requirement for strong suppression of the back lobe is fully satisfied). On the other hand, milder gain roll-offs are obtained for antennas with weak multipath suppression (for which, therefore, the pattern does not drop strongly below the horizon), hence resulting in a better reception of low elevation signals at the price of increased sensitivity to multipath.

### 2.2. Group Delay/Phase Center Variations (GDVs/PCVs)

Group delay variations (GDVs) and phase center variations (PCVs) are the relevant parameters when it comes to the accuracy of distance measurement from receiver to satellite.

For pseudorange measurements, the delay of the signal is the parameter to be “sensed”; therefore, the receiver antenna must make sure to minimize (variations of) its group delay over angle and frequency in order not to introduce errors in the measured delay from the satellite. This parameter is particularly important for timing or aviation applications. In the first case, accurate time estimation is indeed critical and is the aim of the measurement. In the second case, pseudorange measurements are the primary means for PVT determination and are only loosely supported by carrier phase measurements (to reduce the high-frequency noise) due to safety/integrity requirements.

Similarly, for the carrier phase measurements, the phase of the signal carries the information about the distance: a uniform phase pattern of the receiver antenna ensures that no additional phase contribution due to the antenna itself is added to signals from the different satellites and therefore an accurate position can be calculated.

Methods for precisely calculating PCV have been established over the years by the geodetic community: the two most relevant ones are an electromagnetic anechoic chamber calibration and a robot-based calibration [11,12]. The characterization of GDV, on the other hand, still has no widely recognized technique; research is also ongoing in this case to establish the applicability of robot calibration, with the main challenges being the effect of pseudorange noise and multipath errors on the accuracy of the characterization. Chamber calibration appears less prone to these errors and is used by the authors in the following sections.

### 2.3. Multipath Suppression Capability

Finally, as already suggested, the capability to suppress multipath is one of the main characteristics of reference stations: indeed, in applications where accuracy and signal monitoring capabilities are crucial, it is necessary to ensure that measurements are not distorted by reflected signals. These signals bounce over objects, buildings, and terrains in the vicinity of the receiver antenna and then reach the antenna, overlapping with the line-of-sight (LOS) signal and therefore changing the shape of the correlation function, resulting in an error of the estimated delay/phase information from the satellite.

While traditionally, parameters from the world of telecommunications such as the axial ratio or the crosspolar discrimination have been used to estimate the multipath susceptibility [7], it was recently found by the authors that they were not fully describing the actual physics of multipath in GNSS and that parameters more GNSS-related were needed.

Indeed, axial ratio and crosspolar discrimination are calculated by using the two components of the fields (right-handed circular polarization (RHCP) and left-handed circular polarization (LHCP)) in the same direction. This might be useful for communication links but not for multipath phenomena, where the line-of-sight signal from the satellite comes from a given direction {*θ_s_, φ_s_*}, which stands for elevation and azimuth angle from the incoming signal of the satellite. Non-line-of-sight signal, which is bounced on objects around the receiver, arrives from a different direction {*θ_MP_, φ_MP_*}, which stands for elevation and azimuth angle of the incoming MP. In order to take this into account, new parameters have been recently defined by the authors and called “multipath susceptibility indicators” (MPSIs) [13], based on modified versions of the crosspolar discrimination factor (XPD) and of the down-to-up-ratio (DUR).

Different MPSIs can be defined according to the physics of the multipath, e.g., for reflections reaching the receiver antenna from negative or positive elevations, $MPSI_{down}$ or $MPSI_{up}$, respectively.

They are defined as follows [13]:

$$MPSI_{up}=\{\begin{array}{c} \log_{20}\left(-XPD_{max,db}\right)\;if\;XPD_{max,db}<0\\ 0\;if\;XPD_{max,db}>0 \end{array}$$

$$MPSI_{down}=\{\begin{array}{c} \log_{20}\left(-DUR_{max,db}\right)\;if\;DUR_{max,db}<0\\ 0\;if\;DUR_{max,db}>0 \end{array}$$

with

$$XPD_{max}=\frac{max\;(Gain_{LHCP}(\theta_{}>0,\forall \varphi_{}))}{Gain_{RHCP}\left(\theta_{s},\varphi_{s}\right)}{\;}_{}$$

$$DUR_{max}=\frac{max\;(Gain_{TOT}(\theta_{}<0,\forall \varphi_{}))}{Gain_{RHCP}\left(\theta_{s},\varphi_{s}\right)}$$

An MPSI close to 0 means that the multipath is barely suppressed by the antenna; on the other hand, an MPSI of about 0.75 means that the multipath will be attenuated 10 dB with respect to the direct signal, and hence, a medium capability is available. Finally, MPSI values higher than 1 stand for very good multipath suppression given by the antenna. The aforementioned parameters will be used, when possible, in the next Sections to compare the available designs.

## 3. State-of-the-Art of Antennas in Ground Reference Stations

All GNSSs need a monitoring station network as part of the ground segment to monitor the satellite signals and feed observation to control stations to counteract accordingly if needed.

GPS has for instance a monitor station network (MSN) made of 16 stations (6 from the air force and 10 from NGA) [14]. Galileo on the other hand uses a network of Galileo Sensor Stations (GSSs) spread all over the world [15]. In addition, the Galileo Experimental Sensor Stations (GESSs) are used for monitoring the Galileo system performance.

Furthermore, national and international geodetic communities are also maintaining networks of monitoring stations, such as the IGS or the EUREF network [16,17].

Due to the different operators and the evolution over time of the stations, several different antennas are currently operated at the different stations: the most common ones are for sure antennas based on choke-ring technology, capable of strongly limiting multipath from negative elevations. Examples of commercial products making use of this technology are Leica AR25, Novatel GNSS-750, Javad RingAnt-DM, Trimble TRM59800, and TRM59900.

In the following, the performance of commercial antennas is analyzed: if available at DLR, they have been characterized in DLR’s semi-anechoic chamber, as shown in Figure 1. Due to the number of plots, all figures regarding this characterization are included in the Appendix A, Figure A1, Figure A2, Figure A3, Figure A4, Figure A5, Figure A6, Figure A7, Figure A8, Figure A9 and Figure A10.

Most of these antennas are relatively big in size and have a diameter larger than 30 cm. However, recent GNSS antennas with reduced “size, weight, and power cost (SWAP)” have been introduced for high-performance applications: they are addressed in Section 3.2 in order to understand if their performance is also acceptable for reference station applications.

### 3.1. Antennas with Lateral Size Larger Than 30 cm

**Figure 2:**
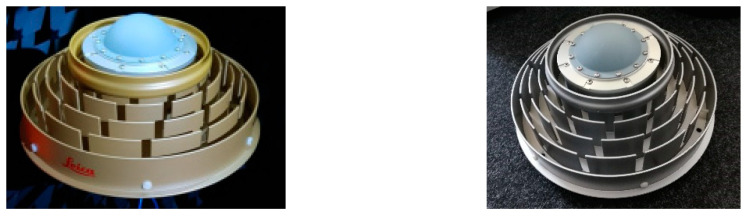
Pictures of Leica AR25 and Novatel GNSS 750.

Among the canonical antennas for reference stations, the first subgroup comprises choke-ring antennas that are typically used as static reference antennas. In this analysis, measurements for the Leica AR25 antenna, the Novatel GNSS-750, and the Javad RingAnt-DM are available, and the first two are shown in Figure 2.

Leica AR25 and Novatel GNSS-750 have both a gain roll-off factor of about 10–11 dB for the L1/E1 band and a factor of 14 dB or 13 dB for the L5/E5 band, respectively. Javad Ring Ant-DM shows slightly higher gain roll-off factors of 16 dB for L1/E1 and for L5/E5 band: signals from low-elevation angles are received with a lower C/$N_{0}$ in this antenna, compared with the former two antennas.

The measured PCV for the frequency bands L1/E1 and L5/E5 are shown in Appendix A. The three choke-ring antennas exhibit a mostly uniform behavior for the PCV over the upper hemisphere, with a maximum range of values of 7.4 mm and 8.2 mm for the Leica AR 25, 8.4 mm and 9.2 mm for the Novatel GNSS 750, and 17.6 mm and 14.5 mm for the Javad RingAnt-DM in the frequency bands L1/E1 and L5/E5, respectively. In general, the PCV has a stable behavior along azimuth and shows variations mostly along the elevation angle, with a “bump” at medium elevations; Javad RingAnt-DM has greater variations among the three.

The GD exhibits absolute mean values around 20–21 ns for Leica AR25 and Novatel GNSS-750 and 18.6 ns for Javad RingAnt-DM, in the L1/E1 band, and values around 19 ns for Leica AR25, 18.8 ns for Novatel GNSS-750, and 21.4 ns for Javad RingAnt-DM in the L5/E5 band. All antennas show a mostly stable behavior of the GDV within the upper hemisphere, with a maximum range of 1.3 ns, 0.8 ns, or 1.2 ns, respectively. Some azimuthal variations are to be seen for Leica AR25 and Javad RingAnt-DM at L1/E1, however, within the range of 0.5 to 0.8 ns.

All three choke-ring antennas achieve a good $MPSI_{up}$, with values above 0.75 in the L1/E1 band from zenith (elevation of 90°) to approximately 15°–20° elevation, enabling the suppression of most multipath from positive elevations. Javad RingAnt-DM is, in this case, performing better than the other antennas, with higher values of MPSI for medium/high elevations, due to its stronger crosspolar discrimination (i.e., low LHCP) throughout the upper hemisphere.

For the L5/E5 band, all three antennas exhibit very good values for $MPSI_{up},$ being close to 1 or even higher at the zenith, becoming lower for decreasing elevation angles, however, still reaching values of approximately 0.5 at the horizon in the L5/E5 band. This behavior can be explained by the low level of LHCP gain in the upper hemisphere for the L5/E5 band, compared to the LHCP gain levels in L1/E1. The $MPSI_{down}$ has a similar behavior for all three choke-ring antennas for both frequency bands. They exhibit good $MPSI_{down}$ values, between 0.8 and 1.0 in higher elevation directions. Additionally, in this case, JavadRingAnt-DM performs slightly better, due to its stronger roll-off, clearly indicating that the antenna was optimized for multipath suppression.

An approach different from choke rings, still leading to similar weight/size, was chosen by Topcon in the PN-A5 antenna, in which instead of choke rings, vertical metallic pins aligned along a semihemispherical ground plane are used to reduce the back radiation. This antenna was not available at DLR: datasheet information [18] shows, however, that the gain roll-off factor is similar to the one from the three choke-ring antennas, being 10 dB for the L1/E1 band and 12 dB for the L5/E5 frequency band.

**Figure 3:**
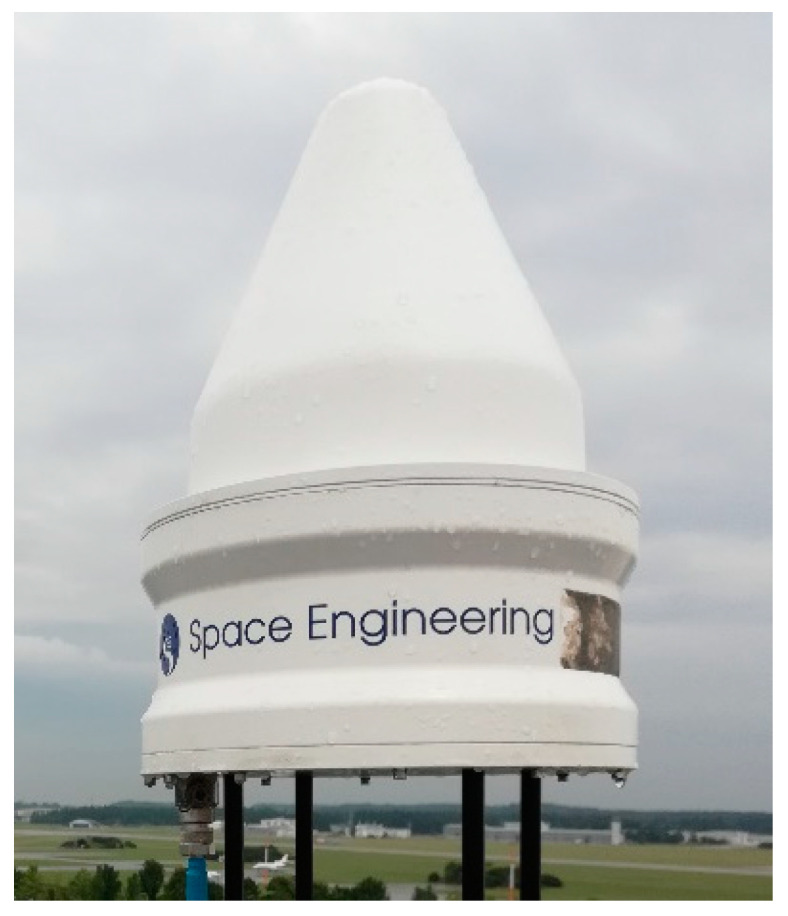
GalExpAnt Antenna from Space Engineering SpA, used for Galileo reference stations.

For Galileo monitoring stations in ESA’s Time and Geodesy Validation Facility (TGVF-X), the Galileo Experimental Antenna (GalExpAnt) from Space Engineering is used (see Figure 3).

Though having a similar size and weight to the choke-ring designs shown before, the technology used in this case is not clearly specified. As later discussed, its performance would not mirror the benefit of choke rings for what concerns multipath suppression. The antenna was characterized by DLR and results can be seen in Appendix A. A gain roll-off factor between 6 and 11 dB for the L1/E1 band and around 11–12 dB for L5/E5 is found. At the L1/E1 band, the RHCP gain does not show minimal variations along the azimuth, indicated by the spread of gain values for different azimuth cuts. At L5/E5, the gain in RHCP is more uniform and changes only slightly along the azimuth.

GalExpAnt exhibits PCV variations within a range of about 10.8 mm for L5/E5 and 22.3 mm for L1/E1. In this case, PCV shows changes along the elevation angle, as was the case for Javad RingAnt-DM or Novatel GNSS-750, in addition to quite significant differences along the azimuth, especially in the L1/E1 frequency band up to 12 mm difference in lower elevations. The GD shows absolute values around 64.8 ns for L1/E1 and 45 ns for the L5/E5 band. The measurements show GDV along elevation with a maximum range of values of 1.9 ns, as well as along the azimuth within maximum a range of 1.2 ns for both L1/E1 and L5/E5 bands.

The $MPSI_{up}$ for this antenna exhibits moderate values around 0.5 in the zenith direction, with values strongly decreasing already at middle elevations, suggesting a poor multipath suppression capability of this antenna at L1/E1. For the L5/E5 band, on the other hand, $MPSI_{up}$ exhibits good values (in the range of 0.6–0.8) in the zenith direction until elevation angles of 15°. Similar behavior can be seen for $MPSI_{down}$. At L1/E1, the values change along the azimuth, and even in the zenith direction, the $MPSI_{down}$ only reaches values of about 0.5, decreasing further for lower elevation angles. In L5/E5, it shows a more uniform behavior along azimuth, with good values of 0.75 at the zenith, but with values below 0.3 already at elevations around 30°, showing also, in this case, a nonoptimal multipath suppression capability for satellites signals coming from low elevations (and multipath from lower hemisphere).

Efforts to reduce the received multipath with the GalExpAnt were, for instance, documented in [19], resulting in an absorbing structure engineered around the antenna and contributing to strongly reducing the amount of multipath impinging on the antenna, in addition to making the installation much more complicated and expensive.

Though GalExpAnt appears particularly not optimal for multipath suppression, similar efforts in reducing errors caused by multipath for ground stations but resulting in complicated designs were also proposed for further antennas from the US side: modifications around a choke-ring antenna have been investigated, e.g., in [20], also in this case strongly complicating the overall antenna structure.

**Figure 4:**
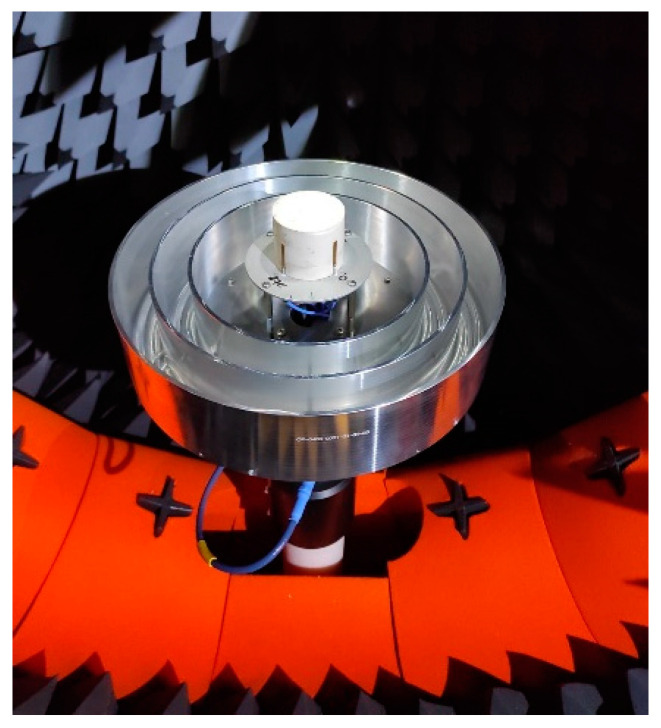
DLR in-house developed antenna with choke rings, as shown in [22], in the anechoic chamber.

A further design, developed internally by DLR, is presented here as well and is shown in Figure 4; this antenna is based on the technology shown in [21] but includes choke rings for multipath suppression as well.

A similar (but reconfigurable) version of the antenna is shown in [22]. The antenna exhibits a gain roll-off factor of 17–18 dB for L1/E1 and L5/E5, hence similar to Javad RingAnt-DM. The pattern is for both frequency bands quite uniform along the azimuth. Its PCV values vary in a maximum range of 17.5 mm for L5/E5 and 14.7 mm for the L1/E1 band, being slightly higher than the PCV of Javad RingAnt-DM. This is due to the relatively high absolute values close to elevation angles of 0°, whereas in medium/higher elevation angles the PCV exhibits lower variations than Javad RingAnt-DM. The mean GD for this antenna shows very low values, compared to the aforementioned antennas, with absolute values around 2.5 ns for L1/E1 and 6.6 ns for L5/E5. This is due to the fact that the antenna (at the time of measurement) was still passive, i.e., was not integrating active components, strongly contributing to the mean value of group delay. An active version of the design is currently being manufactured. Variations of GD over angle are in the range of 0.7 ns at both frequency bands.

$MPSI_{up}$ at L1/E1 and L5/E5 appears particularly good, with values around 1.0 in zenith direction and bigger than 0.3 for elevations higher than 15°. $MPSI_{down}$, similar to the COTS choke-ring antennas analyzed before, has values close to 1 at zenith and bigger than 0.3 for elevations higher than 15°.

In conclusion, the antennas analyzed in this subgroup show high performance in terms of pattern uniformity, gain roll-off, stable PCV/GDV, and very good multipath suppression. However, differences in performance can be observed among the different products, such that a careful antenna selection (considering the parameters that will be more relevant for the installation scenario) is strongly advisable before installation.

### 3.2. Antennas with Lateral Size Smaller Than 30 cm

**Figure 5:**
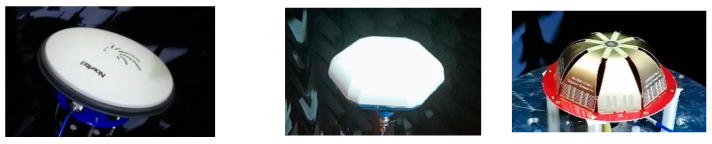
Novatel 703-GGG, Navxperience Nav3G+C, and Tallysman Verostar VSE6028, all in the anechoic chamber at DLR.

The next subgroup consists of three high-precision GNSS antennas that are smaller in size and lighter in weight than the previously considered antennas, as they are not equipped with a choke ring or a similar structure to reduce multipath. Measurements were performed at DLR and can be compared more in detail in this analysis for Novatel 703-GGG, NavXperience Nav3G+C, and Tallysman Verostar VSE6028, shown in Figure 5.

Novatel 703-GGG [23] shows a mostly uniform behavior of the RHCP gain and a gain roll-off factor of 11–12 dB in L1/E1 and between 8 and 10 dB in L5/E5, with slight variations along the azimuth. Nav3G+C [24] has a gain roll-off factor between 11 and 14 dB for L1/E1 and 9–10 dB for L5/E5 and shows small gain variations along the azimuth. Tallysman VSE 6028 [25] has a smaller gain roll-off, of only about 6–9 dB in L1/E1 and 7–10 dB in L5/E5.

Regarding PCV, Novatel 703-GGG shows very low PCV, with a maximum value range of 2.2 mm for the L5/E5 band and 4.5 mm for the L1/E1 band. PCV appears stable throughout the whole upper hemisphere. Nav3G+C exhibits PCV with a maximum range of 10.3 mm for L5/E5 and 5.7 mm for the L1/E1 band and small changes along elevation. Tallysman VSE6028, on the other hand, has a PCV in the range of 18.1 mm in L5/E5 and 8.1 in the L1/E1 band with a slight azimuth dependence.

When looking at group delay, Novatel 703-GGG shows GD mean values around 18.7 ns for L1/E1 and 20.6 ns for L5/E5, being rather stable within the upper hemisphere (i.e., with a range of 0.6 ns). Nav3G+C has absolute GD values around 7.7 ns and 9.0 ns for L1/E1 and L5/E5, respectively, and is also quite uniform within the upper hemisphere (with a maximum variation range of 0.7 ns). Tallysman has absolute GD values of 14 ns at L5/E5 and 28 ns at L1/E1, with a variation range up to 1.4 ns along elevation.

Both Novatel 703-GGG and Tallysman VSE 6028 show very good behavior for the $MPSI_{up}$, with values around 1 for the whole upper hemisphere for L1/E1 and between 0.7 and 0.8 for L5/E5. $MPSI_{up}$ for Nav3G+C is also very similar to the one of Novatel 703-GGG antenna with good values between 0.8 and 1.0 for the whole hemisphere at L5/E5, while it has only good performance (though still comparable to the choke rings) for L1/E1. The $MPSI_{down}$ exhibits for both Novatel 703-GGG and Nav3G+C antennas good values in zenith direction, while goes below 0.3 for elevations smaller than 20°. The multipath susceptibility for Tallysman VSE6028 is poor at both bands, with $MPSI_{down}$ values bigger than 0.3 already reached at elevations of 30° due to the lower back lobe suppression and roll-off.

In conclusion, smaller high-performance antennas are available on the market capable of strongly minimizing PCV/GDV and achieving a very broad beamwidth. In this case, multipath suppression is often not one of the main design goals, and therefore, poor performance can be obtained by some designs. However, some commercial products, such as Novatel 703-GGG, appear to have a very good balance between size and multipath suppression capability.

A summary of the performance achievable by the different antennas is given in Table 1: a qualitative assessment of their performance in terms of multipath is also given with a “simplified” scale of + and -. Moreover, data for further commercial antennas, as obtained by their datasheets, are also included in the table for completeness.

The results shown till now demonstrate that the bigger (choke-ring) antennas clearly exhibit the best performance in terms of high gain roll-off factor and multipath suppression, particularly for the lower hemisphere, while also ensuring low and stable PCV and GDV. Smaller high-precision antennas also showed that a very good performance can be achieved also with smaller sizes: very good performance in terms of PCV/GDV stability can be obtained also in this case. Indeed, the absence of the choke rings is beneficial in achieving pattern uniformity.

However, as expected, the capability of smaller antennas to suppress multipath is, in general, poorer, in particular for reflections coming from the lower hemisphere. Good results can, however, be obtained when the requirement for multipath suppression is taken into consideration in the antenna design by using smaller and simplified multipath limitation structures, such as the Pinwheel technology used in Novatel 703-GGG, leading to only slightly worse performance than the ones obtained with much bigger and heavier choke-ring antennas.

## 4. High-Performance Antennas for Space Applications

The application of GNSS in space is even more demanding than for Earth applications; its use becomes, however, in such contexts almost necessary to precisely determine the position, velocity, and time of a spacecraft. Apart from the aforementioned antenna performance parameters, such as multiple frequency band capability, the purity of the polarization, and stable PCV and GDV, there are several additional aspects that have to be considered for spaceborne antennas that affect their performance quite significantly. The used materials need to be space qualified to make sure that they can handle the different conditions of the space environment (high-temperature fluctuation, radiation, or outgassing) [39]. Moreover, GNSS antennas on satellites are mounted in close vicinity to other antennas for communication or instrumental purposes. Their electromagnetic interaction shall be properly accounted for in order to minimize intersystem interference. The choice of an optimal location for the antenna on the satellite platform is, therefore, the first countermeasure to consider [39]. Furthermore, mass, size, and power consumption are strictly limited onboard a satellite, as higher mass or required power directly relates to higher launch/operational costs. Therefore, it is desired to keep the mass and size as low as possible and to achieve the high efficiency of the antenna.

LEO satellites already use GNSS antennas to determine their position, velocity, and time. One recent example is the ESA mission Sentinel-6, which is equipped with spaceborne GNSS receivers from RUAG Space [40] for precise orbit determination (POD). Herefore, precise multifrequency PEC antennas from RUAG and multi-GNSS receivers are used. Another example is the CubeSat mission Bobcat-1 from the Ohio State University [41], launched in 2020, also equipped with a multifrequency multi-GNSS receiver and a commercial GNSS antenna from Antcom (Antcom G5), typically used for airborne applications. Moreover, GNSS is also used on the international space station (ISS), where multipath suppression capability is especially significant [42,43].

Additionally, satellites flying at higher altitudes are more and more using GNSS signals, using the space service volume (SSV) [44]. For this purpose, spaceborne GNSS receivers specifically targeting GEO applications are available on the market.

While the former examples show the feasibility of using GNSS in space, the exploding number of satellites, following the new space paradigm, also makes a strong and growing interest in high-performance spaceborne GNSS antennas clearly foreseeable.

While for smaller CubeSats or small satellites on low budgets, small and low-cost antennas with a moderate performance are often used, satellites with the need to perform a more precise position determination have to use higher-performing antennas. Novel applications can make use of recent developments and the now achievable PNT accuracy levels, which enables the possibility of creating reference stations in orbit. These would have the benefit of not having atmospheric effects on the signals and much fewer distortions from the environment, i.e., multipath from far objects, unless the one caused by the spacecraft itself, allowing a “cleaner,” more accurate monitoring and observation of the GNSS signals, with strong improvements for the whole satellite navigation system [6].

The “ideal antenna” for ground-based reference stations is described in Section 2. Some of the requirements are also valid for spaceborne applications in LEO, for instance, with respect to PCV/GDV and multipath suppression. Different from ground-based antennas on Earth, antennas in LEO need to be able to receive GNSS signals coming from very low or even negative elevation angles from GNSS satellites being on the opposite side of Earth. Hence, having a higher gain for small negative elevation angles (i.e., a very low gain roll-off) can be needed in this case. In GEO, on the other hand, the platform is situated in an orbit above the GNSS constellations, which means that GNSS signals can only be received from satellites on the opposite side of Earth, transmitting signals past Earth. The signals travel a larger distance, and therefore, a higher and more directive gain pattern is needed. Both for LEO and GEO applications, the antenna will still need to have a low GDV and PCV in order to enable precise POD. Multipath suppression capability can also play a role for instance to minimize the impact of reflections coming from solar panels or other platform structures, such as on the ISS.

Size and weight are also driving parameters for space applications and need to be properly minimized. With this in mind, some selected antennas, commercially available, for space applications are compared in Table 2 in terms of size, weight, operating frequency bands, and gain roll-off factor, as obtainable from the respective datasheet. Unfortunately, information about PCV or GDV for these antennas is not always available.

Various antennas operable at L1/E1 band are available on the market (i.e., from the companies ISIS [45], ANYWAVES [46], SkyFoc Labs [47], Space Quest [48], NewSpace Systems [49], RUAG Space [50]). Being small in size and lightweight makes them suitable for smaller CubeSats but not for precise dual-frequency applications. The majority of them are microstrip patch antennas and have a gain roll-off factor of 7–8 dB. RUAG PEC antenna uses a patch-excited cup technology, which is higher in weight and size but achieves a gain roll-off factor of about 13 dB and likely also a superior multipath suppression.

Antennas designed for use in GEO orbits are, as already mentioned, fulfilling different requirements, with the clearest one being the need for a more focused, highly directive beam. Examples of this class of antennas are RUAG Space’s PEC GEO antenna and Fraunhofer’s SUGA antenna (see Table 2).

**Figure 6:**
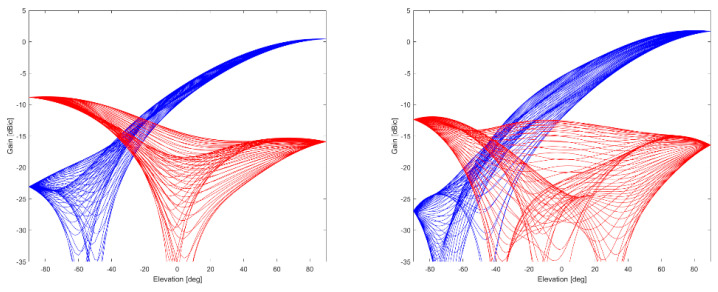
Gain in dBic (RHCP in blue, LHCP in red, different lines represent different azimuth values with a resolution of 5.625 deg) over elevation angle at L1/E1 (**left**) and L5/E5 (**right**) of ANYWAVES GNSS All-Bands Antenna (data provided by manufacturer).

**Figure 7:**
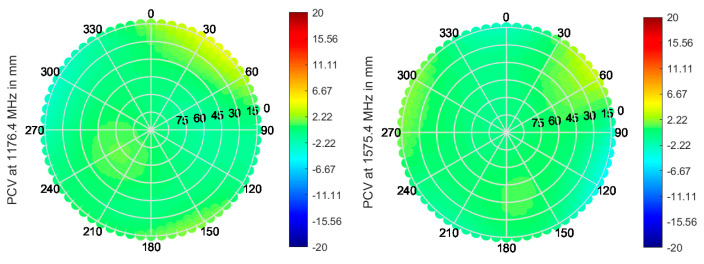
PCV in the upper hemisphere at L1/E1 (**left**) and L5/E5a (**right**) for the ANYWAVES GNSS All-Bands Antenna, with data provided by manufacturer.

**Figure 8:**
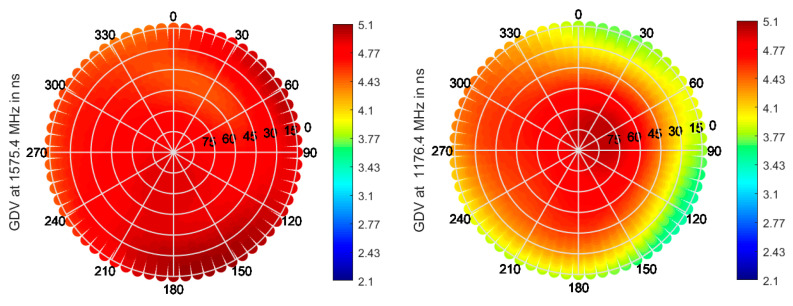
GDV in the upper hemisphere at L1/E1 (**left**) and L5/E5a (**right**) for the ANYWAVES GNSS All-Bands Antenna, with data provided by manufacturer.

Multi-frequency capable antennas are also transitioning from research (e.g., Fraunhofer GNSS CubeSat antenna) to becoming more and more commercially available. For instance, ANYWAVES All-Bands GNSS Antenna, which was recently space qualified, is based on printed antenna technology and can cover L5/E5, L2, E6, and L1/E1 bands. Data provided by the manufacturer are shown in Figure 6, Figure 7 and Figure 8.

A roll-off of about 8–10 dB can be seen for both bands, as well as very stable PCV and GDV, indicating the good performance of the antenna.

Different multifrequency antennas are available from RUAG: on the one side, Patch Excited Cup (PEC) antennas are available with or without choke rings (named corrugations by RUAG): the latter achieves a gain roll-off of about 10–14 dB. Moreover, a quadrifilar helix antenna is available, exhibiting a very low gain roll-off factor of about 0 dB for L1/E1, L5/E5, respectively. Unfortunately, no measurement data were available to the authors, and therefore, only information from datasheet [50] can be analyzed.

## 5. Exemplary Performance

The former sections described the state of the art in terms of antennas for very demanding applications such as reference stations and shown the achievable performance in terms of electromagnetic indicators, either as declared by the manufacturer or, when possible, as measured at DLR premises.

For some of the antennas available at DLR, it was also possible to perform a comparative measurement campaign in terms of GNSS measurements, in order to compare the actual GNSS performance that these antennas would achieve.

In order to do so, the following antennas were considered: Leica AR25, Novatel GNSS 750, Galileo Experimental Antenna, Javad RingAnt-DM, DLR-in-house design, NavXperience 3G+C, Tallysman VSE6028.

**Figure 9:**
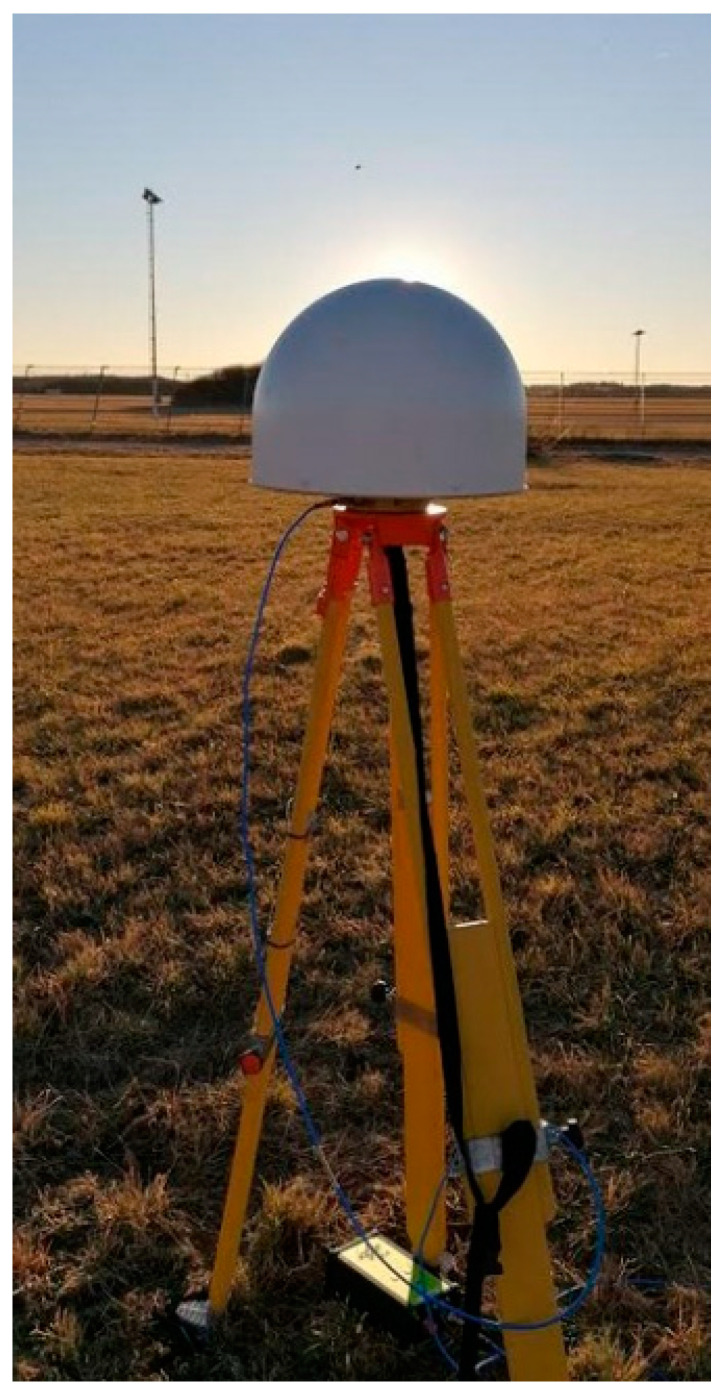
Leica AR25R4 antenna placed on a tripod in DLR campus field for GNSS measurement.

The antennas were tested at DLR campus in Oberpfaffenhofen, Germany, positioned on a tripod at a height of 150 cm in an open field (Figure 9), where bigger obstacles (buildings, etc.) are more than 80 m away and only affect lower elevations (up to about 15 deg). Only one obstacle, namely, the fence dividing the field from the nearby airport, is known to cause punctual multipath at higher elevations (around 60 deg elevation) [13]: its effect will be also visible in these measurement campaigns. Measurements were collected for at least 12 h for each setup.

The antennas were evaluated in terms of C/$N_{0}$ (i.e., carrier-to-noise ratio, directly affected by the gain characteristics of the antenna) and 100 s smoothed multipath and noise errors over elevation (calculated through dual-frequency code-minus-carrier methods [54]). The smoothing is used to reduce the high-frequency noise, allowing a better comparison between the different antennas. Please note once more that different C/$N_{0}$ maximum levels are mostly related to the LNAs of the antennas (i.e., passive antennas have lower maximum C/$N_{0}$ than active antennas); it is, therefore, useful to focus on the C/*N*_0_ spread over azimuth/roll-off over elevation more than the actual maximum value achieved.

**Figure A11:**
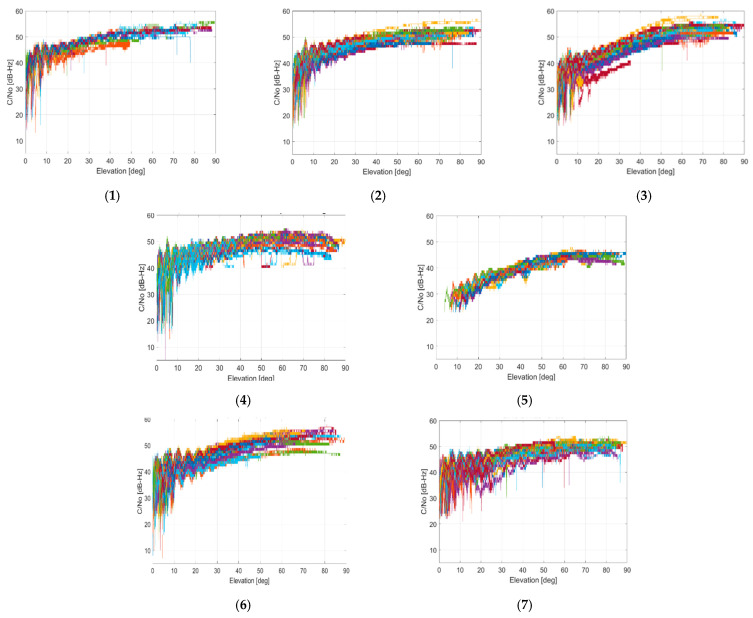
C/N0 at L1/E1 for the antennas measured at DLR; from left to right, from top to bottom: (**1**) Leica AR25 R4; (**2**) Novatel GNSS-750; (**3**) Javad RingAnt-DM; (**4**) Space Engineering GalExpAnt; (**5**) DLR internal design; (**6**) Navxperience Nav3G+C; (**7**) Tallysman VSE6028 VeroStar. Different colors indicate different PRNs.

**Figure A12:**
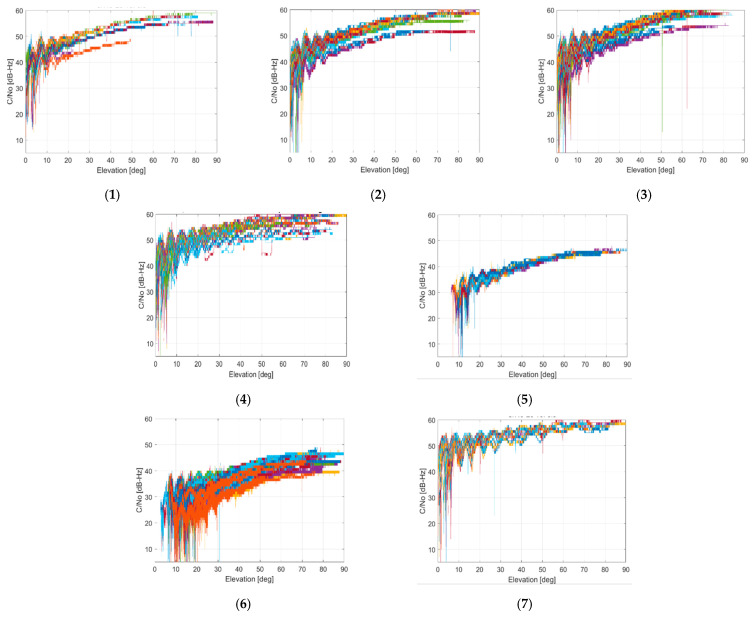
C/N0 at L5/E5a for the antennas measured at DLR; from left to right, from top to bottom: (**1**) Leica AR25 R4; (**2**) Novatel GNSS-750; (**3**) Javad RingAnt-DM; (**4**) Space Engineering GalExpAnt; (**5**) DLR internal design; (**6**) Navxperience Nav3G+C; (**7**) Tallysman VSE6028 VeroStar. Different colors indicate different PRNs.

**Figure A13:**
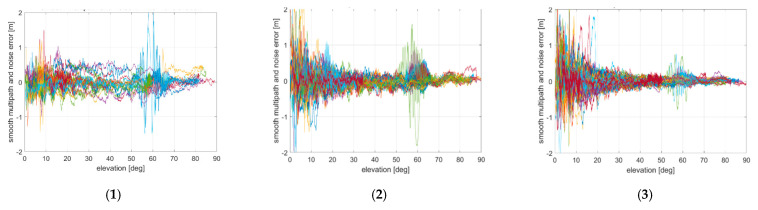

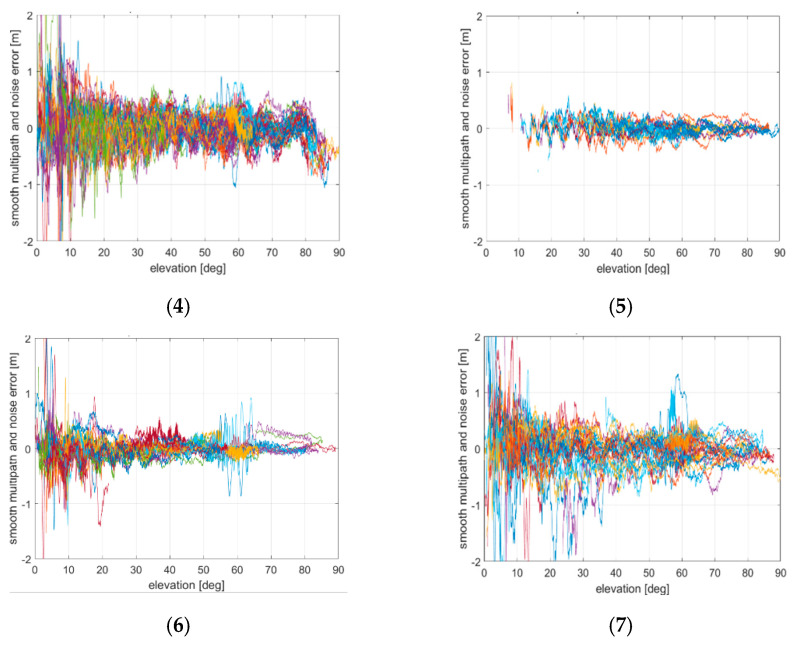
The 100 s smoothed multipath (estimated through CMC) at L1/E1 for the antennas measured at DLR; from left to right, from top to bottom: (**1**) Leica AR25 R4; (**2**) Novatel GNSS-750; (**3**) Javad RingAnt-DM; (**4**) Space Engineering GalExpAnt; (**5**) DLR internal design; (**6**) Navxperience Nav3G+C; (**7**) Tallysman VSE6028 VeroStar. Different colors indicate different PRNs.

**Figure A14:**
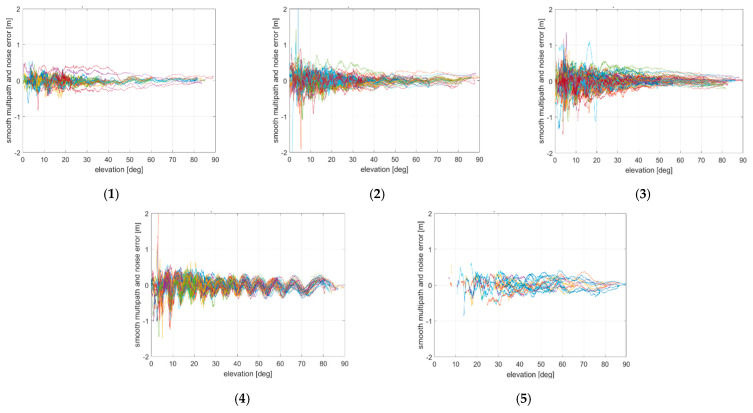

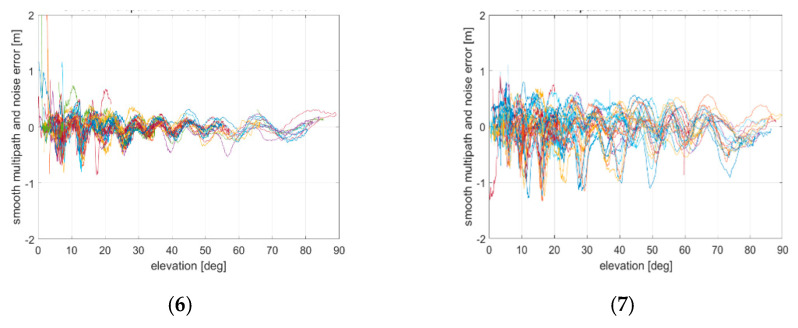
The 100 s smoothed multipath (estimated through CMC) at L5/E5a for the antennas measured at DLR; from left to right, from top to bottom: (**1**) Leica AR25 R4; (**2**) Novatel GNSS-750; (**3**) Javad RingAnt-DM; (**4**) Space Engineering GalExpAnt; (**5**) DLR internal design; (**6**) Navxperience Nav3G+C; (**7**) Tallysman VSE6028 VeroStar. Different colors indicate different PRNs.

The results from Figure A11, Figure A12, Figure A13 and Figure A14 (in Appendix A) show indeed the different levels of performance that can be obtained by the antennas under test from GNSS perspective in a “reference-station-like” scenario.

**Figure 10:**
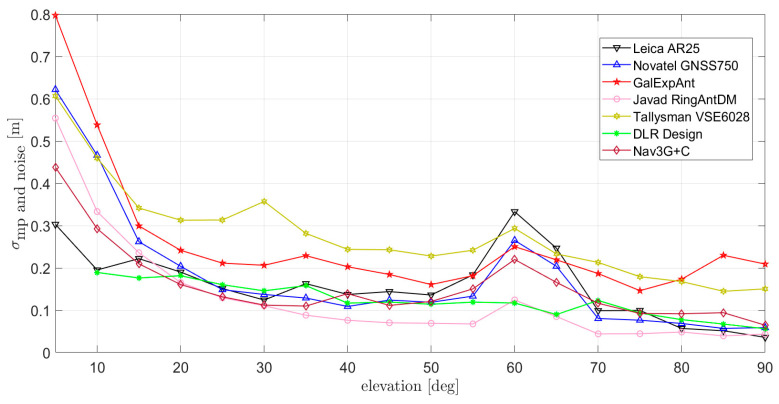
RMS of 100 s smoothed multipath (estimated through CMC) at L1/E1 for the antennas measured at DLR: (1) Leica AR25 R4; (2) Novatel GNSS-750; (3) Javad RingAnt-DM; (4) Space Engineering GalExpAnt; (5) DLR internal design; (6) Navxperience Nav3G+C; (7) Tallysman VSE6028 VeroStar.

Indeed, the differences in the antenna design impact the stability and values of C/$N_{0}$ (for instance, by enabling or not to receive signals from low elevations with enough C/$N_{0}$ level), as well as the amount of multipath received. In particular, looking at Figure A13 and Figure A14, it is clearly visible how the low elevation multipath and the punctual multipath from the airport fence, resulting in a peak around 60 deg elevation, are differently suppressed by the different antennas. In order to summarize these results, the RMS per elevation bin is calculated and reported in Figure 10. A superior multipath suppression can be observed for the DLR design and the Javad RingAntDM, while poor multipath suppression is recorded for the Tallysman VSE6028, as already expected from the MPSI characterization in Section 3.

The results shown in this Section are obtained in a low-multipath installation (Figure 10).

**Figure 11:**
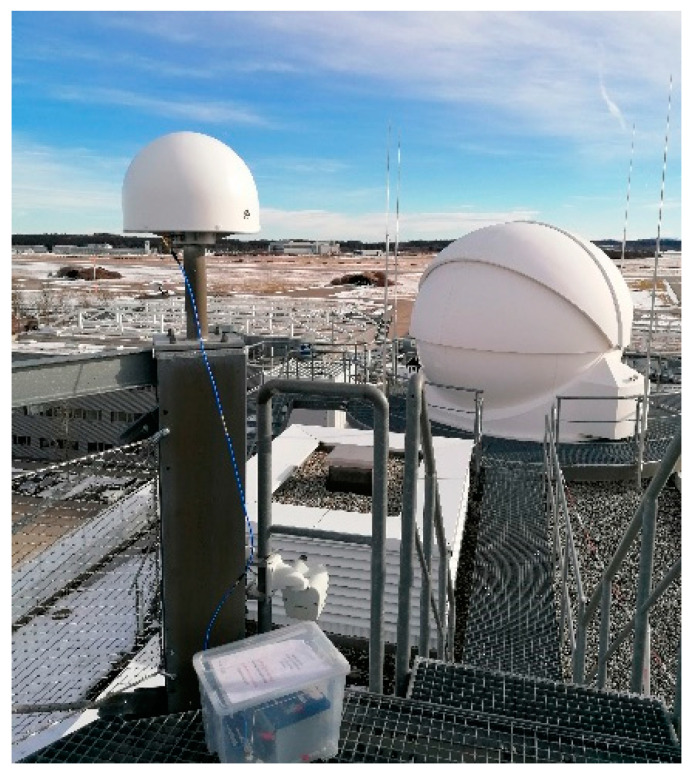
Example of reference antenna mounted in a more challenging environment, in this case, the rooftop of DLR’s Institute of Communications and Navigation.

**Figure 12:**
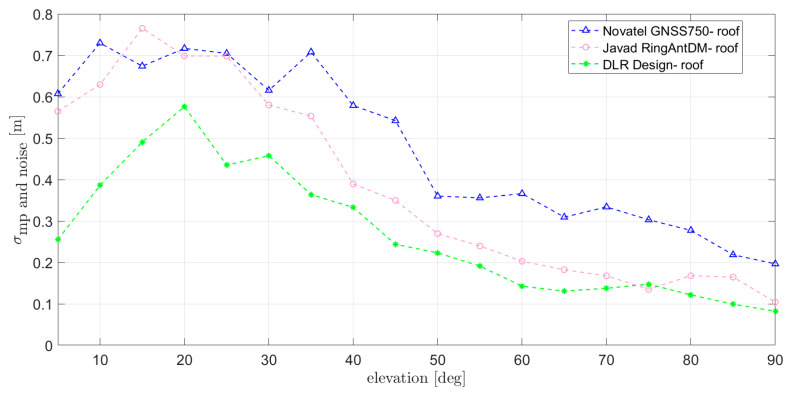
RMS of 100 s smoothed multipath (estimated through CMC) at L1/E1 for the antennas installed on the roof of DLR’s Institute of Communications and Navigation.

However, reference antennas sometimes also need to be installed on less multipath-benign scenarios, such as shown in Figure 11. Though the results are by nature very installation dependent, it is nevertheless useful to provide insight into the multipath performance to be expected in those cases. In order to do so, three of the antennas previously analyzed (Novatel GNSS750, Javad RingAntDM, and DLR design) could be installed on the roof of DLR’s Institute of Communications and Navigation (Figure 11). The obtained results are shown in Figure 12: an overall performance degradation is observed with respect to the “open field” case, due to the multiple reflecting objects in the vicinity. Additionally, in this case, DLR design and Javad RingAntDM perform best and show the lowest RMS multipath errors.

## 6. Open Research Issues

Former sections showed the achievable performance for current antennas for high-performance applications. It was shown how important it is to characterize their performance parameters properly, as they will affect the power level and quality of the received signals. In general, the importance of choosing proper antennas for the specific installation should be emphasized: in complicated installation scenarios, such as rooftops, or in situations where the minimization of multipath effects is a key factor, a strong emphasis should be given to antennas providing very good multipath suppression, often coupled with strong gain roll-off factors. Moreover, for precise applications, clear preference should be given to antennas exhibiting minimal GDV/PCV, such that the antenna itself contributes minimally to the overall error. In applications where size and cost are constrained a specific trade-off should be found, taking into consideration the specific requirements.

It was also seen how state-of-the-art designs for reference station antennas tend to have large size and weight and that recent new designs have been suggested and come to the market with smaller SWAP-C factors. Miniaturization is for sure an open research issue that will also need to be tackled in the future; indeed, smaller SWAP-C models while retaining optimal performance (mostly in terms of multipath suppression, with choke rings being the most established and heavy countermeasure for that) will need to be achieved.

A further open research challenge is the addition of interference suppression capability to the design of reference station antennas. As a matter of fact, reference station performance can degrade due to both non-human factors, such as installation-related multipath and unintentional or intentional interference (both in terms of jamming and spoofing): this threat appears to gain momentum in the last years, with disruptions to ground stations documented already in 2013 [55], happening more and more often in the latest years.

A powerful countermeasure to interference (both for detection and suppression) is represented by multiantenna systems, capable of dynamically placing nulls (i.e., strongly suppressing the received power) in the direction of the interference. Adaptive multiantenna systems can also be valuable with respect to multipath suppression, using also in this case nulls in the direction of multipath.

Such systems have been widely demonstrated for robust applications in the world of research (see, e.g., [56,57]), and products are already commercially available (see, e.g., Novatel GAJT product family).

Research is, however, still needed in order to harmonize robustness and precision requirements. The adaptive nulling creates a variable pattern, with nulls provoking changes in phase and amplitude of the pattern also in their vicinity, hence possibly affecting the performance of the antenna for precision applications. Work is currently being performed in this respect at multiple institutions worldwide.

## 7. Conclusions

In this work, a review of GNSS antennas as sensors for high-end satellite navigation applications, in particular targeting reference station applications and space applications, was performed. The most relevant performance indicators for such kinds of applications were identified and used for comparing multiple commercial antennas, making use of anechoic chamber measurement data. Field tests were also performed, validating the former results. It was demonstrated that antenna design directly impacts the achievable performance and that the antenna to be used in a specific scenario should be selected considering which performance is most critical for the envisaged use.

Furthermore, GNSS antennas for space use were also evaluated and achievable performance was shown: due to the current new space trend and the strong increase in the number of satellites being launched, it is expected that high-performance space-qualified antennas will also see a strong increase in the near future. Open research issues were discussed and the main trends identified, namely, the quest for miniaturization (without sacrificing performance) and for the addition of interference suppression capabilities, for instance, by making use of multiantenna technologies.

## Figures and Tables

**Figure 1:**
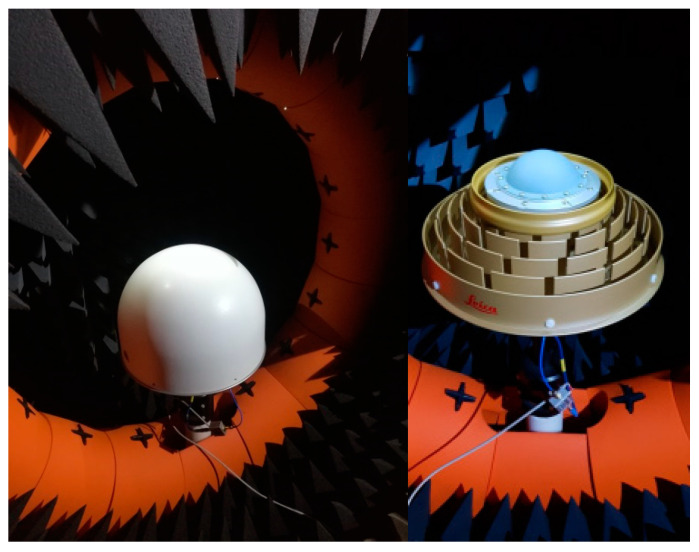
Novatel GNSS 750 (**left**) and Leica AR25 (**right**) antenna as measured at DLR’s near field antenna test facility in Oberpfaffenhofen.

**Table 1:** Comparison of GNSS Rx antennas for ground applications.

Antenna	Technique	Size	Weight	Frequency Bands	Gain Roll-Off Factor	$\mathrm{Multipath}\;\mathrm{Suppression}\;MPSI_{up}$ $/MPSI_{down}$	Phase Center Variation PCV (Maximum Range in mm)	Group Delay Variation GDV (Maximum Range in ns)
**Leica AR25** [26]	Chokering	380 × 380 × 200 mm^3^	7.6 kg	L5/E5ab, L2, E6, L1/E1	**L5/E5: 14 dB** **L1/E1: 10 dB**	**L5/E5: +++/+** **L1/E1: +/+**	**L5/E5: 8 mm** **L1/E1: 7 mm**	**L5/E5: 0.8 ns** **L1/E1: 1.3 ns**
**Novatel GNSS 750** [27]	Chokering	380 × 380 × 200 mm^3^	7.6 kg	L5/E5ab, L2, E6, L1/E1	**L5/E5: 13 dB** **L1/E1: 11 dB**	**L5/E5: +++/+** **L1/E1: +/+**	**L5/E5: 9 mm** **L1/E1: 8 mm**	**L5/E5: 0.4 ns** **L1/E1: 0.8 ns**
**Javad RingAnt DM** [28]	Chokering	380 × 380 × 138 mm^3^	4.4 kg	L5/E5ab, L2, E6, L1/E1	**L5/E5: 16 dB** **L1/E1: 16 dB**	**L5/E5: +++/++** **L1/E1: ++/++**	**L5/E5: 15 mm** **L1/E1: 18 mm**	**L5/E5: 0.8 ns** **L1/E1: 1.2 ns**
**Space Engineering GalExpAnt**	Not specified	294 × 294 × 459 mm^3^	~16 kg	E5ab, E6, E1	**L5/E5: 11–12 dB** **L1/E1: 6–11 dB**	**L5/E5: +/o** **L1/E1: -/- -**	**L5/E5: 11 mm** **L1/E1: 22 mm**	**L5/E5: 1.9 ns** **L1/E1: 1.7 ns**
**DLR antenna**	Chokering	~350 × 350 × 300 mm^3^	~8 kg	L5/E5ab, L2, E6, L1/E1	**L5/E5: 17–18 dB** **L1/E1: 17–18 dB**	**L5/E5: ++/++** **L1/E1: ++/++**	**L5/E5: 18 mm** **L1/E1: 15 mm**	**L5/E5: 0.6 ns** **L1/E1: 0.7 ns**
**Septentrio PolaNt Chokering B3/E6** [29]	Chokering	376 × 376 × 350 mm^3^	5.0 kg	L5/E5ab, L2, E6, L1/E1	L5/E5: 11 dB L1/E1: 11 dB	n.a.	n.a.	n.a.
**Trimble GNSS v2 Chokering antenna** [30]	Chokering	380 × 380 × 146 mm^3^	4.3 kg	L5/E5ab, L2, E6, L1/E1	L5/E5: - L1/E1: -	n.a.	n.a.	n.a.
**Topcon CR G5** [31]	Chokering	380 × 380 × 155 mm^3^	4.9 kg	L5/E5ab, L2, E6, L1/E1	L5/E5: 16.5 dB L1/E1: 13 dB	n.a.	n.a.	n.a.
**Topcon PN A5** [18]	Vertical dipoles	380 × 380 × 262 mm^3^	6.7 kg	L5/E5ab, L2, E6, L1/E1	L5/E5: 12 dB L1/E1: 10 dB	n.a.	n.a.	n.a.
**Leica AR10** [32]	Planar structure with large GND plane	240 × 240 × 140 mm^3^	1.1 kg	L5/E5ab, L2, E6, L1/E1	L5/E5: n.a. L1/E1: n.a.	n.a.	n.a.	n.a.
**Novatel 704-X** [33]	NoVAtel’s patented Pinwheel${\;}^{®}$ technology	185 × 185 × 69 mm^3^	0.468 kg	L5/E5ab, L2, E6, L1/E1	L5/E5: 11 dB L1/E1: 14 dB	n.a.	n.a.	n.a.
**Novatel 703-GGG** [23]	Pinwheel${\;}^{®}$	185 × 185 × 69 mm^3^	0.500 kg	L5/E5ab, L2, E6, L1/E1	**L5/E5: 8–10 dB** **L1/E1: 11–12 dB**	**L5/E5: +++/-** **L1/E1: +++/+**	**L5/E5: 2 mm** **L1/E1: 4 mm**	**L5/E5: 0.6 ns** **L1/E1: 0.3 ns**
**Novatel GNSS 850** [34]	Multi-point feeding network	176 × 176 × 55 mm^3^	0.507 kg	L5/E5ab, L2, E6, L1/E1	L5/E5: 12 dB L1/E1: 10 dB	n.a.	n.a.	n.a.
**Tallysman Verostar VSE6028** [25,35]	VeroStar${\;}^{\mathrm{TM}}$ technology	106 × 106 × 39 mm^3^	0.080 kg	L5/E5ab, L2, E6, L1/E1	**L5/E5: 7–10 dB** **L1/E1: 6–9 dB**	**L5/E5: ++/- -** **L1/E1: ++/-**	**L5/E5: 18 mm** **L1/E1: 8**	**L5/E5: 1.1 ns** **L1/E1: 1.4 ns**
**Navxperience Nav3G+C** [24,36]	Mobile	172 × 172 × 121 mm^3^	0.384 kg	L5/E5ab, L2, E6, L1/E1	**L5/E5: 9–10 dB** **L1/E1: 11–14 dB**	**L5/E5: +++/-** **L1/E1: +++/+**	**L5/E5: 10 mm** **L1/E1: 6 mm**	**L5/E5: 0.3 ns** **L1/E1: 0.7 ns**
**Septentrio/TallysmanVeraPhase 6000** [37]	VeraPhase${\;}^{®}$	167 × 167 × 175 mm^3^	0.820 kg	L5/E5ab, L2, E6, L1/E1	L5/E5/L2: 11 dB L1/E1: 13 dB	n.a.	n.a.	n.a.
**Septentrio PolaNt-x MC** [38]	Mobile	190 × 190 × 73 mm^3^	0.450 kg	L5/E5ab, L2, L1/E1	L5/E5: 11 dB L1/E1: 11 dB	n.a.	n.a.	n.a.

Data reported in BOLD are based on measurements performed at DLR.

**Table 2:** Comparison of GNSS Rx antennas for space applications.

Antenna	Technique	Size	Weight	Frequ. Bands	Gain Roll-Off Factor	Multipath Suppression	Phase Center Variation PCV	Group Delay Variation GDV
**ISIS GNSS L1/E1** [45]	Patch antenna	70 × 70 × 15 mm^3^	18 g	L1/E1	L1/E1: ~7 dB	n.a.	n.a.	n.a.
**ANYWAVES GNSS L1/E1** [46]	Patch antenna	68 × 70 × 12.1 mm^3^	86 g	L1/E1	L1/E1: ~8 dB	n.a.	n.a.	n.a.
**SkyFoc Labs GPS-L1 piPATCH-MAX** [47]	Patch antenna	74 × 74 × 13 mm^3^	89 g	L1	L1/E1: n.a	n.a.	n.a.	n.a.
**Space Quest ANT-GPS L1** [48]	Patch antenna	17.5 × 52.8 × 52.8 mm^3^	82 g	L1 (+L2)	L1/E1: 7 dB (to 10° ele.)	n.a.	n.a.	n.a.
**New Space Systems NANT-PTCL1** [49]	Patch antenna	54 × 54 × 14.1 mm^3^	80 g	L1	L1/E1: -	n.a.	n.a.	n.a.
**RUAG PEC L1/E1** [50]	Patch Excited Cup	144 × 144 × 35 mm^3^	220 g	L1/E1	L1/E1: ~13 dB	n.a.	n.a.	n.a.
**Fraunhofer GNSS Cubesat** [51]	Multifeed 3D technology	100 × 83 × 10 mm^3^	20 g	L5/E5ab, L2, E6, L1/E1	L5/E5: 7 dB L1/E1: ~11 dB	n.a.	n.a.	n.a.
**ANYWAVES GNSS all bands** [52]	Printed antenna	90 × 90 × 15 mm^3^	123 ± 4 g	L5/E5ab, L2, E6, L1/E1	L5/E5: 8–10 dB L1/E1: 7–13 dB	n.a.	L5/E5: <8 mm L1/E1: <9 mm	L5/E5: 1.5 ns L1/E1: 0.7 ns
**RUAG PEC (wo. corrugation)** [50]	Patch Excited Cup	160 × 160 × 55 mm^3^	325 g	L5/E5ab, L2, L1/E1	L5/E5: 13 dB L1/E1: 18 dB	n.a.	n.a.	n.a.
**RUAG PEC (w. corrugation)** [50]	Patch Excited Cup	200 × 200 × 87 mm^3^	735 g	L5/E5ab, L2, L1/E1	L5/E5: 14 dB L1/E1: 18 dB	n.a.	n.a.	n.a.
**RUAG Helix** [50]	Quadrifilar helix	90 × 90 × 410 mm^3^	0.815 kg	L5/E5ab, L2, L1/E1	L5/E5: 1 dB L1/E1: 0 dB	n.a.	n.a.	n.a.
**RUAG PEC GEO** [50]	Patch Excited Cup	239 × 239 × 179 mm^3^	715 g	L1/E1	n.a.	n.a.	n.a.	n.a.
**Fraunhofer SUGA** [53]	One arm helix	250 × 250 × 50 mm^3^	<1000 g	L5/E5	n.a.	n.a.	n.a.	n.a.

**Figure A1:**
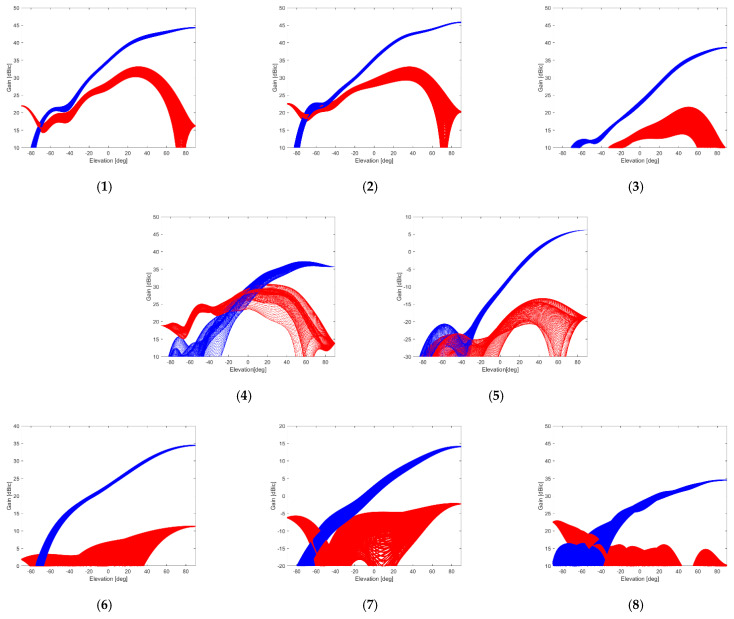
Gain in dBic (RHCP in blue, LHCP in red; different lines represent different azimuth values with a resolution of 2 deg) at L1/E1 central frequency of the antennas measured at DLR; from left to right, from top to bottom: (**1**) Leica AR25 R4, (**2**) Novatel GNSS-750, (**3**) Javad RingAnt-DM; (**4**) Space Engineering GalExpAnt; (**5**) DLR internal design; (**6**) Novatel 703-GGG; (**7**) NavXperience Nav3G+C; (**8**) Tallysmann VSE6028 VeroStar.

**Figure A2:**
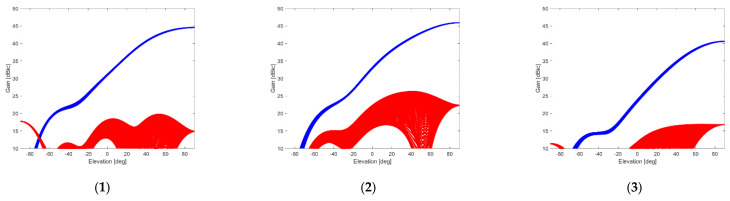

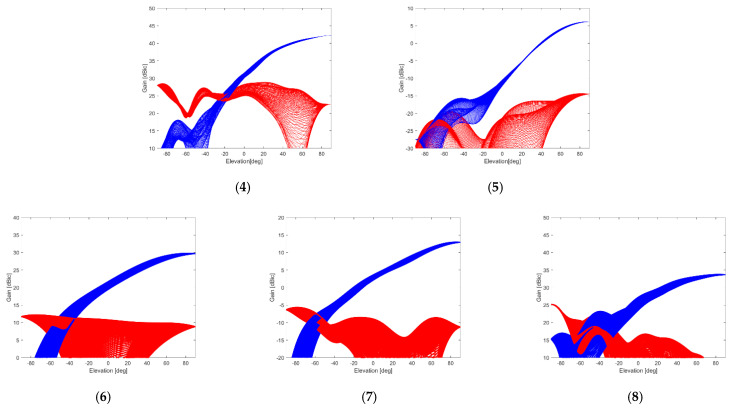
Gain in dBic (RHCP in blue, LHCP in red; different lines represent different azimuth values with a resolution of 2 deg) at L5/E5a central frequency of the antennas measured at DLR; from left to right, from top to bottom: (**1**) Leica AR25 R4; (**2**) Novatel GNSS-750; (**3**) Javad RingAnt-DM; (**4**) Space Engineering GalExpAnt; (**5**) DLR internal design; (**6**) Novatel 703-GGG; (**7**) Navxperience Nav3G+C; (**8**) Tallysmann VSE6028 VeroStar.

**Figure A3:**
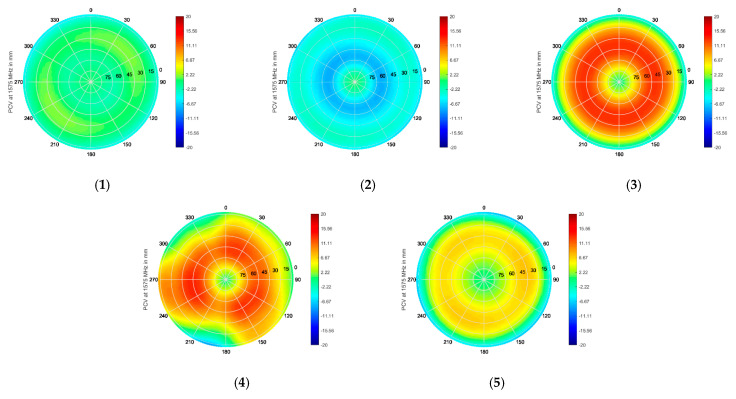

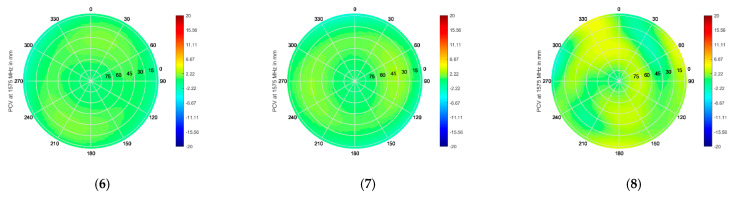
Normalized phase center variations (PCVs) in mm at L1/E1 central frequency of the antennas measured at DLR; from left to right, from top to bottom: (**1**) Leica AR25 R4; (**2**) Novatel GNSS-750; (**3**) Javad RingAnt-DM; (**4**) Space Engineering GalExpAnt; (**5**) DLR internal design; (**6**) Novatel 703-GGG; (**7**) Navxperience Nav3G+C; (**8**) Tallysmann VSE6028 VeroStar.

**Figure A4:**
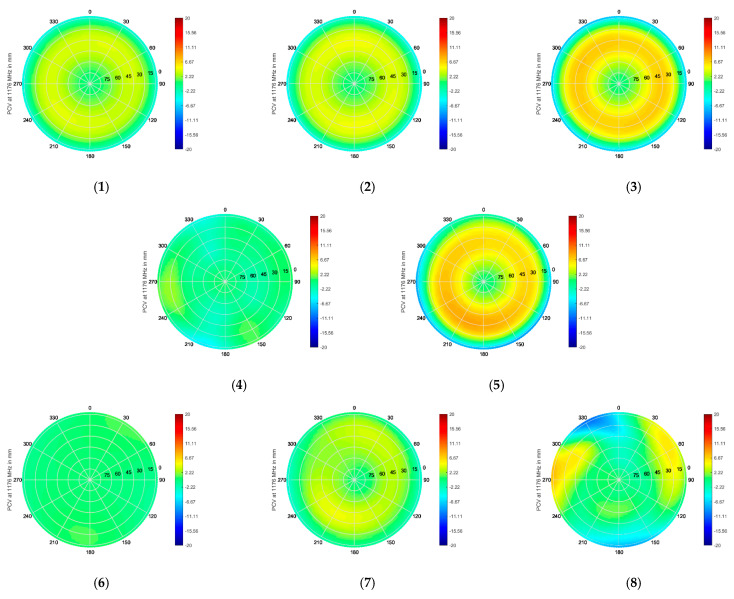
Normalized phase center variations (PCVs) in mm at L5/E5a central frequency of the antennas measured at DLR; from left to right, from top to bottom: (**1**) Leica AR25 R4; (**2**) Novatel GNSS-750; (**3**) Javad RingAnt-DM; (**4**) Space Engineering GalExpAnt; (**5**) DLR internal design; (**6**) Novatel 703-GGG; (**7**) Navxperience Nav3G+C; (**8**) Tallysmann VSE6028 VeroStar.

**Figure A5:**
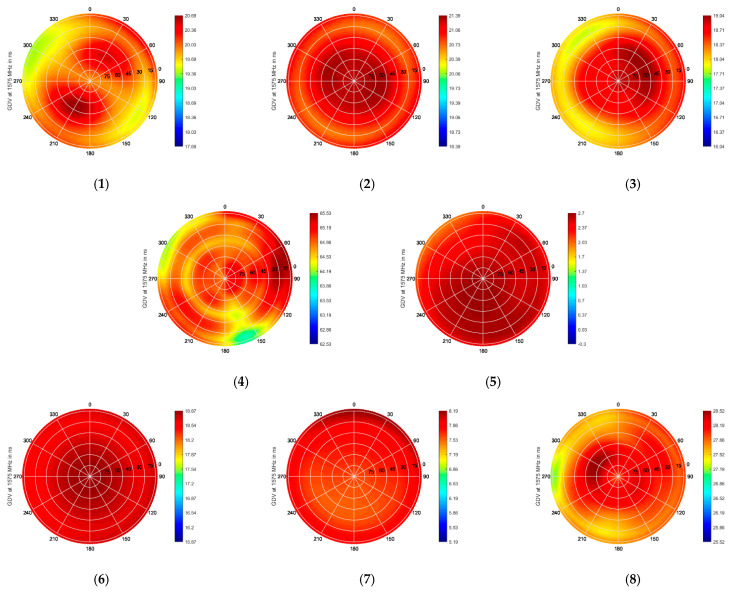
Group delay in ns at L1/E1 central frequency of the antennas measured at DLR; from left to right, from top to bottom: (**1**) Leica AR25 R4; (**2**) Novatel GNSS-750; (**3**) Javad RingAnt-DM; (**4**) Space Engineering GalExpAnt; (**5**) DLR internal design; (**6**) Novatel 703-GGG; (**7**) Navxperience Nav3G+C; (**8**) Tallysman VSE6028 VeroStar.

**Figure A6:**
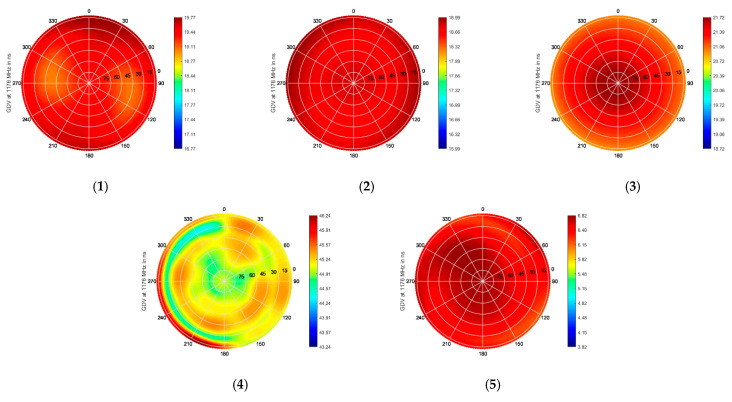

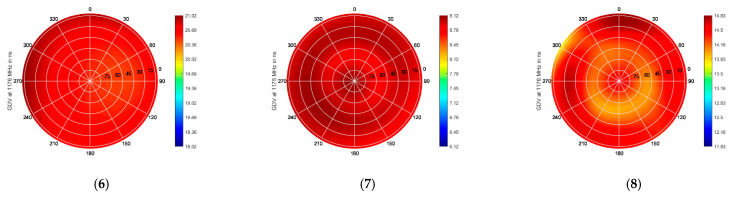
Group delay in ns at L5/E5a central frequency of the antennas measured at DLR; from left to right, from top to bottom: (**1**) Leica AR25 R4; (**2**) Novatel GNSS-750; (**3**) Javad RingAnt-DM; (**4**) Space Engineering GalExpAnt; (**5**) DLR internal design; (**6**) Novatel 703-GGG; (**7**) Navxperience Nav3G+C; (**8**) Tallysman VSE6028 VeroStar.

**Figure A7:**
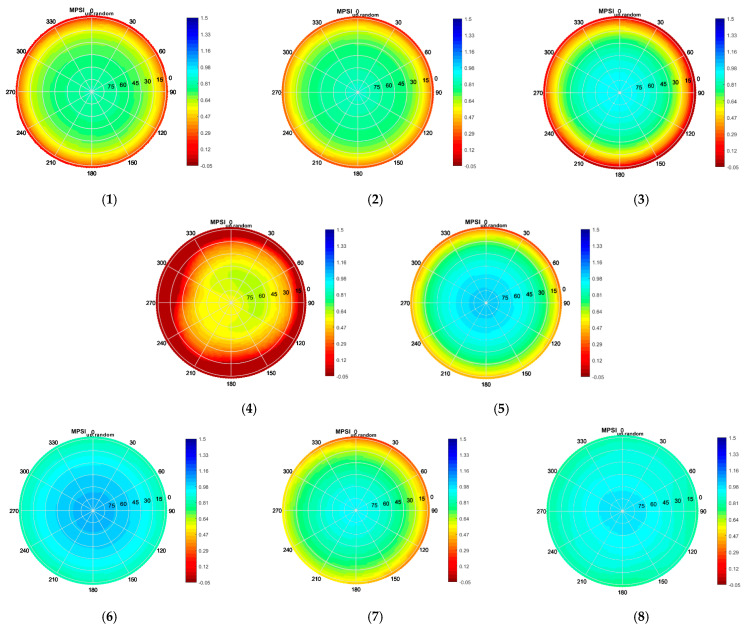
MPSI_up_ at L1/E1 central frequency of the antennas measured at DLR; from left to right, from top to bottom: (**1**) Leica AR25 R4; (**2**) Novatel GNSS-750; (**3**) Javad RingAnt-DM; (**4**) Space Engineering GalExpAnt; (**5**) DLR internal design; (**6**) Novatel 703-GGG; (**7**) Navxperience Nav3G+C; (**8**) Tallysman VSE6028 VeroStar.

**Figure A8:**
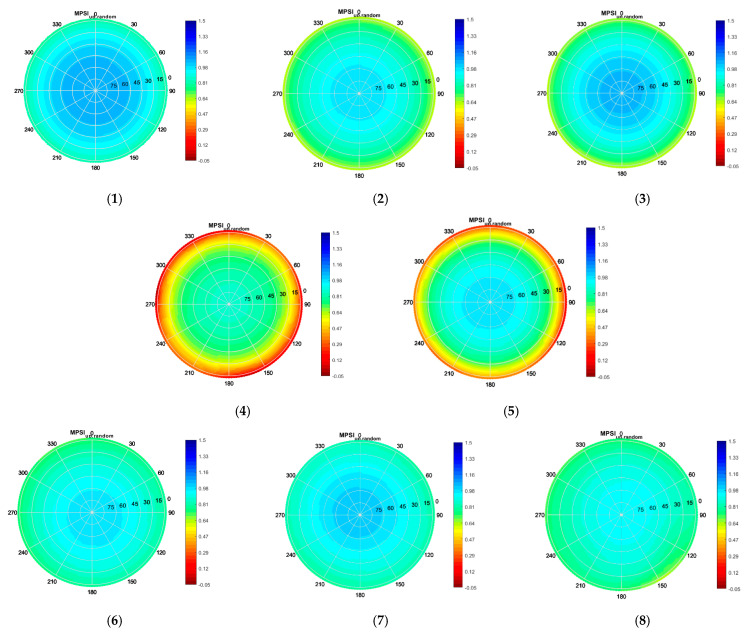
MPSI_up_ at L5/E5a central frequency of the antennas measured at DLR; from left to right, from top to bottom: (**1**) Leica AR25 R4; (**2**) Novatel GNSS-750; (**3**) Javad RingAnt-DM; (**4**) Space Engineering GalExpAnt; (**5**) DLR internal design; (**6**) Novatel 703-GGG; (**7**) Navxperience Nav3G+C; (**8**) Tallysman VSE6028 VeroStar.

**Figure A9:**
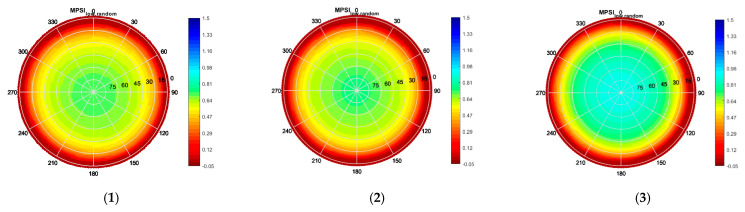

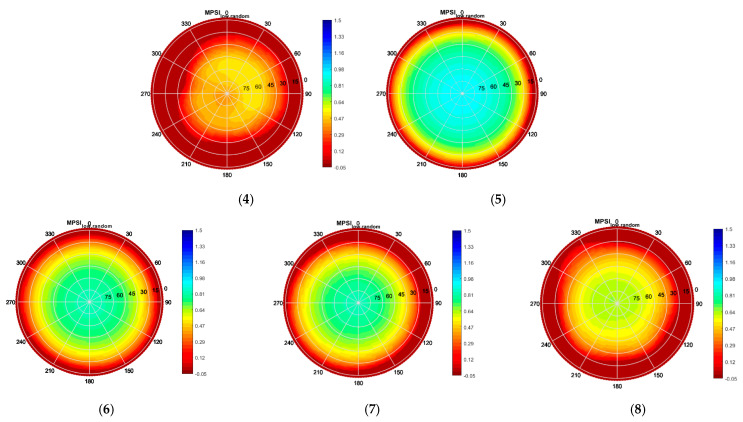
MPSI_down_ at L1/E1 central frequency of the antennas measured at DLR; from left to right, from top to bottom: (**1**) Leica AR25 R4; (**2**) Novatel GNSS-750; (**3**) Javad RingAnt-DM; (**4**) Space Engineering GalExpAnt; (**5**) DLR internal design; (**6**) Novatel 703-GGG; (**7**) Navxperience Nav3G+C; (**8**) Tallysman VSE6028 VeroStar.

**Figure A10:**
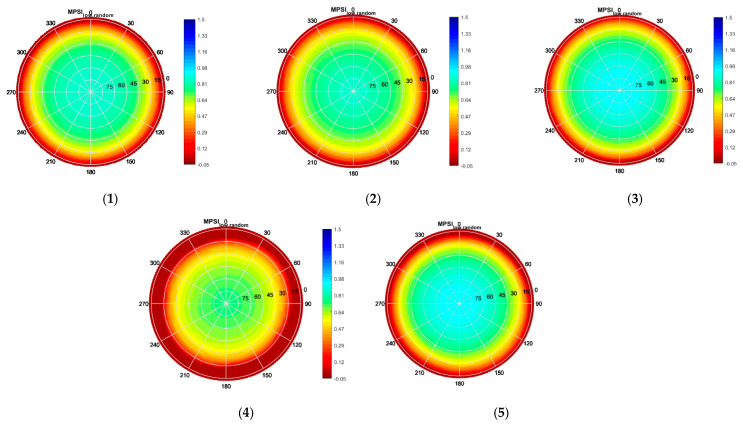

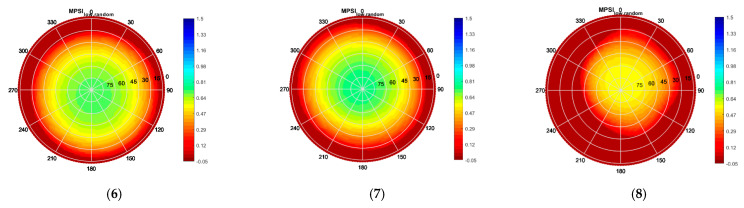
MPSI_down_ at L5/E5a central frequency of the antennas measured at DLR; from left to right, from top to bottom: (**1**) Leica AR25 R4; (**2**) Novatel GNSS-750; (**3**) Javad RingAnt-DM; (**4**) Space Engineering GalExpAnt; (**5**) DLR internal design; (**6**) Novatel 703-GGG; (**7**) Navxperience Nav3G+C; (**8**) Tallysman VSE6028 VeroStar.

## Appendix A

The plots for the different antennas exhibit always a gain range of 40 dB along the *y*-axis, though having different maximum gain values. This is due to the fact that some antennas are passive and some are active with different LNA gains.

The plots for the different antennas exhibit always a gain range of 40 dB along the *y*-axis, though having different maximum gain values. This is due to the fact that some antennas are passive and some are active with different LNA gains.
